# Exploring the Determinants of Chinese Tourists’ Shopping Behavior During Shopping Tourism in Korea

**DOI:** 10.3390/bs14100971

**Published:** 2024-10-20

**Authors:** Qizhen Dong, Shuai Ling, Can Zheng, Yan Hu, Feng Lin, Seul Ki Lee

**Affiliations:** 1 School of Transportation, Weifang Vocational College of Food Science and Technology, Anqiu 262100, China; dongqizhen1991@hotmail.com; 2 Department of Design and Manufacturing Engineering, Jeonbuk National University, Jeonju 54896, Republic of Korea or shuailing@jbnu.ac.kr (S.L.); canzheng@jbnu.ac.kr (C.Z.); 3 College of Hospitality and Tourism Management, Sejong University, Seoul 05006, Republic of Korea; huyan0909@naver.com (Y.H.); linfeng@sju.ac.kr (F.L.); 4 Tourism Industry Data Analytics Lab (TIDAL) and College of Hospitality and Tourism Management, Sejong University, Seoul 05006, Republic of Korea

**Keywords:** shopping behavior, modified theory of planned behavior, shopping tourism in Korea, Chinese tourists

## Abstract

This study aimed to examine the factors influencing Chinese tourists’ shopping behavior during shopping tourism in Korea using a theoretical framework based on the modified theory of planned behavior (TPB). A quantitative research method was used; the questionnaire for this study was posted on Wenjuanxing for data collection and 269 valid questionnaires were analyzed in September 2024 using partial least-squares structural equation modeling (PLS-SEM). The results of analyzing the 269 questionnaires showed that attitude, conformity, perceived behavioral control, and perceived quality of goods influenced shopping intention. However, face consciousness did not affect shopping intentions. Additionally, Chinese tourists’ shopping intentions influenced their shopping behavior during shopping tourism in Korea. The results of this study reveal the potential mechanisms of the influence of shopping intentions with conformity (a factor of Chinese-subjective norms) and the perceived quality of goods (a new factor) in shopping behavior, and they provide theoretical guidance and appropriate marketing strategies for companies in the Korean tourism field.

## 1. Introduction

Due to the rapid growth of global tourism, Chinese tourists have emerged as a significant tourist group worldwide [1]. In 2019 alone, a staggering 154.6 million Chinese citizens traveled abroad, ranking first in the world [2]. One topic of great concern is that shopping has emerged as a crucial component of Chinese outbound tourism. Shopping tourism is a form of travel where the primary goal is to purchase multiple items that are difficult to find outside their place of origin and are appealing to tourists, which means that shopping is regarded as the “primary goal” in shopping tourism [3,4]. Additionally, Li et al. based their study on the finding that for Chinese tourists visiting other countries, shopping is the most significant activity [5]. As Tsang et al. argue, Chinese tourists’ primary reason for visiting Hong Kong is shopping [6]. However, there is currently a dearth of research on Chinese tourists’ shopping behavior, and many studies concentrate on Chinese tourists’ purchases of specific goods when traveling abroad [4,7].

The Korean Wave is spreading throughout Asia, including China, which is close to Korea, as well as other nations [8]. Consequently, the Korean Wave has caused an increase in the number of foreign tourists—particularly Chinese tourists—visiting Korea [9]. The number of international visitors to Korea reached its highest level in 2019 with 17,502,756 people, and Chinese tourists held the top spot with 6,709,451 people [10]. Additionally, the Korean Wave has had a significant impact on Chinese tourists’ intention to shop when visiting Korea [11]. Yoon pointed out that shopping is the main reason that Chinese tourists come to Korea [12]. On the other hand, Seo et al. emphasized the significance of Japanese tourists’ shopping behaviors while visiting Korea [13]. Nguyen et al. investigated Vietnamese tourists’ preferences for shopping tourism in Korea [14]. As a result, shopping tourism is one of the primary strategic initiatives of nations when encouraging sustainable and high-quality tourism [15]. Korea is striving hard to develop as a shopping and tourism destination that is famous worldwide [16].

Although many studies have been conducted on the overseas shopping behavior of Chinese tourists, previous studies have been general and lacked specificity [17], especially since there have been relatively few in-depth investigations into Chinese tourists’ shopping behavior in Korea. The theory of planned behavior is a commonly used research method for predicting consumers’ behavioral intentions. However, whether the theory of planned behavior developed in the United States is applicable to Chinese tourists is worth further empirical research [18,19,20]. Therefore, the following research questions are posed:

(1) What factors affect Chinese tourists’ shopping intentions while shopping in Korea?
(2) Does shopping intention affect shopping behavior within the modified theory of planned behavior?

To address the problems mentioned above, we constructed a conceptual model of Chinese tourists’ shopping behavior based on the modified theory of planned behavior. The conceptual model of the modified theory of planned behavior in this study replaces the subjective norms of the original theoretical model of the theory of planned behavior with Chinese-subjective norms—the face consciousness and conformity variables—and secondly, a new variable, perceived quality of goods, is added to the original model of the theory of planned behavior to form the final framework model. The hypotheses were tested by using partial least-squares structural equation modeling (PLS-SEM), determining the influence factors of Chinese tourists’ shopping intention, better understanding Chinese tourists’ behavioral motivations for shopping tourism in Korea, enhancing the image and status of Korea on the global shopping tourism market [21], and improving the satisfaction of shopping tourism for Chinese tourists [22]. On the other hand, this study aimed to identify the factors that influence Chinese tourists’ shopping intentions while shopping in Korea, thereby determining the relationship between shopping intentions and shopping behaviors, and expanding the application areas of the modified theory of planned behavior [23]. Additionally, this study provides insight into Chinese tourists’ shopping behavior during shopping tourism in Korea [24], and the most innovative point was the use of the modified theory of planned behavior through the PLS-SEM method, which has not been carried out in previous studies. On the other hand, some practical implications for the tourism industry, retailers, policymakers, and the design of tourism products in Korea were outlined to provide directions for improving marketing strategies or services related to Chinese tourists’ shopping behavior and boosting the Korean economy [25].

Therefore, this study is organized into the following sections: Section 1 introduces the status of Chinese tourists’ shopping behavior and shopping tourism in Korea, the problems in Chinese tourists’ shopping behavior and shopping tourism in Korea, and the purpose of this study. Section 2 presents the research hypotheses. Furthermore, based on a literature review, the shortages and limitations in previous studies are shown. Section 3 discusses the research strategy, measurement items, survey development, data collection method, and analytical methods. Section 4 outlines the results. Section 5 concludes by highlighting possible discussions on the outcomes and presenting some implications.

## 2. Literature Review

### 2.1. Shopping Tourism

Due to the discovery of the connections between purchases, motivations, and the choice of destination, the topic of “shopping tourism” has recently been a hot topic in academic studies and has become more and more important in the field of tourism [26]. Shopping tourism is travel in which the primary goal is to purchase multiple items that are difficult to find outside their place of origin and are appealing to tourists [17].

Moreover, Liberato et al. claimed that shopping tourism is a major economic engine for tourism and commercial growth in several nations [26]. Wijayanti et al. found that shopping tourism has become one of the most popular kinds of tourism, and it creates economic chances for locals and has a positive influence on their well-being [17,27]. On the other hand, as Tomori argues, shopping has become a more and more important part of travel and accounts for a significant amount of tourists’ expenditures [28]. This means that shopping has developed into a determining factor when selecting a travel destination and the primary purpose for traveling [26].

According to Timothy’s research, shopping tourism is growing in Europe, North America, and South America, and is having a significant impact on the economy [29]. However, there are few studies on shopping tourism in Asia, especially in Korea; thus, in this study, we selected Korea as the research nation. Lee et al. investigated whether the perceived tour quality had a positive effect on tourist satisfaction in Korea [30].

Additionally, Lo and Qu found that the quality of goods has a significant impact on shopping behavior, which further shows that the theory of planned behavior applies to shopping tourism [31]. Unfortunately, there is only a limited amount of research on the theory of planned behavior in the academic literature on shopping tourism. Hence, this study was based on the modified theory of planned behavior to research Chinese tourists’ shopping behavior in Korea.

### 2.2. Modified Theory of Planned Behavior

The theory of planned behavior is an expanded version of the theory of reasoned action [32]. However, a rising number of studies have discovered that attitude and subjective norms cannot control actual behavior, and additional influencing factors must be added to the theory of reasoned action [33]. Therefore, Ajzen formalized a theory of planned behavior by adding a new variable, perceived behavioral control, to the basic theory of reasoned action [18]. According to the definition of the theory of planned behavior, attitude, subjective norms, and perceived behavioral control act together as factors of behavioral intention that affect behavior.

However, Seong and Hong proposed that the theory of planned behavior needs to be expanded to increase its explanatory power [34]. Hence, many studies have used the modified theory of planned behavior, especially in the field of consumer behavior within tourism [35].

#### 2.2.1. Attitude 

Ajzen proposed that attitude is a behavioral or emotional tendency to respond positively or negatively to a thing, situation, feeling, idea, or person [18], meanwhile Fishbein revealed that a person’s attitude toward engaging in a certain action might be positive or negative [36]. When an activity is positively evaluated, people will wish to engage in it. In this study, attitude refers to the psychological experience or evaluation of the shopping behavior of Chinese tourists while on a shopping tour in Korea. Chinese tourists are more likely to show a positive shopping intention when they have a positive evaluation of shopping during shopping tourism in Korea. However, Bangun and Handra argued that attitude cannot influence shopping behavior directly, as it is always influenced through intention [37]. Therefore, we propose the following hypothesis:

**Hypothesis** **1** **(H1).** *Attitude positively influences shopping intentions during shopping tourism in Korea.*

#### 2.2.2. Subjective Norms

A subjective norm is a perception or point of view about the beliefs of others that affects one’s intention to engage in a behavior or not [18]. Normative belief indicates the extent to which one is motivated to adopt the viewpoint of others in connection to the behavior that they will perform [38]. Moreover, normative belief refers to expectations that are derived from people or organizations that are viewed as influential, such as parents, partners, close friends, or others, depending on the behavior involved [18]. Chinese scholar Li et al. argued that face consciousness and conformity represent the subjective norms in Chinese culture [39]. Thereby, a suitable model for Chinese cultural characteristics appeared and was confirmed. Hence, the subjective norms of this study involved three variables of face consciousness and conformity, which represented the subjective norms of Chinese culture. Moreover, Li et al. stated that face consciousness and conformity, as subjective norms, cannot directly affect behavior [39].

Zhang et al. argued that Chinese tourists often place a high value on their group’s reputation and self-worth [40]. Face consciousness is referred to as “the social anchoring of oneself in the eyes of others or groups” [41].

Loss of face means losing essential social respect in one’s own eyes and others. In other words, losing one’s respectable and good social dignity in the sight of others leads to feelings of humiliation and awkwardness [42,43]. Hsu and Huang regarded face consciousness as an essential variable in understanding Chinese tourists’ consumption behavior [23]. Hence, the definition of face consciousness in this study refers to a perception of one’s own status and reputation. Measurement items of face consciousness have been adopted in prior studies, and the measurement scales were modified [38]. We present the following hypothesis:

**Hypothesis** **2-1** **(H2-1).** *Face consciousness positively influences shopping intentions during shopping tourism in Korea.*

Zhou et al. argued that conformity can be defined as someone’s intention to be affected by others and has a significant impact on behavioral intentions [44]. Susilawati and Nova found that conformity is a conscious or unconscious effort to alter one’s attitudes, behaviors, or views to fit a group [45]. Hence, as Pool and Schwegler argued, people tend to look to others for guidance when faced with uncertainties, especially in China, which focuses on a harmonious culture and group orientation [46]. Accordingly, the definition of conformity in this study refers to others in the group who are also shopping, which means that if others in the group are not shopping, the subject is also not shopping, and we expect the following relationship to hold:

**Hypothesis** **2-2** **(H2-2).** *Conformity (others in the group are also shopping) positively influences shopping intentions.*

#### 2.2.3. Perceived Quality of Goods

Perceived quality of goods can be defined as a consumer’s belief in the excellence of a product that is perceivable. Moreover, the perceived quality of goods is determined by the customer’s perceptions of the total experience of using that good and is not limited to the attributes of a specific one [47]. Since fake goods have flooded the domestic market in China, whenever Chinese tourists can travel abroad, they choose to buy high-quality foreign goods [48]. Therefore, it is not difficult to find that Chinese tourists are very concerned about the perceived quality of goods. Meanwhile, with the popularity of high-quality Korean goods, many fake goods posing as Korean goods are circulating in the domestic market in China. Thus, Chinese tourists are willing to buy high-quality goods when shopping in Korea. Additionally, this study, to further understand the shopping behavior of Chinese tourists when shopping in Korea, introduced a new variable—perceived quality of goods—as this can make the modified model more comprehensive and convincing [49]. On the other hand, the perceived quality of goods is a visible and tangible variable that is different from attitude and subjective norms. Therefore, based on the theoretical and empirical studies above, we expect the following relationships to hold:

**Hypothesis** **3-1** **(H3-1).** *The perceived quality of goods positively influences shopping intentions during shopping tourism in Korea.*

**Hypothesis** **3-2** **(H3-2).** *The perceived quality of goods positively influences shopping behavior during shopping tourism in Korea.*

#### 2.2.4. Perceived Behavioral Control

The theory of reasoned action was expanded by Ajzen to include a new construct called “perceived behavioral control” as an additional determinant of both intention and behavior [18]. It refers to the perceptions of consumers of their power to complete a given activity. Specifically, Farooq et al. stated that when an activity is viewed as being very simple to complete, there is a great likelihood that people will carry it out [50]. Hence, the definition of perceived behavioral control in this study refers to the resources, situations, and possibilities that Chinese tourists are able to perceive, as well as how easy or difficult shopping is in Korea [51]. We propose the following hypotheses:

**Hypothesis** **4-1** **(H4-1).** *Perceived behavioral control positively influences shopping intentions during shopping tourism in Korea.*

**Hypothesis** **4-2** **(H4-2).** *Perceived behavioral control positively influences shopping behavior during shopping tourism in Korea.*

#### 2.2.5. Shopping Intentions and Behavior

Azhar et al. argued that intention is a cognitive representation of one’s willingness to carry out a certain behavior, which implies that the most reliable predictor of actual behavior is intention, and an actual behavior can be realized if someone has the intention to engage in a behavior [32]. On the other hand, Brown et al. stated that consumers who express a willingness to purchase will have a greater percentage of actual purchases than consumers who express no desire to buy [52]. Moreover, Ajzen argued that intention has a significant influence on behavior [18]. Shopping intentions are defined in this study as the possibility of Chinese tourists performing a shopping behavior while shopping in Korea. Shopping behavior is defined in this study as Chinese tourists performing actual buying behavior while shopping in Korea [51]. As a result, based on the theoretical and empirical studies above, we hypothesize the following:

**Hypothesis** **5** **(H5).** *Shopping intentions positively influence shopping behavior during shopping tourism in Korea.*

## 3. Methodology

### 3.1. Questionnaire

There were three sections to the official questionnaire. The respondents were first given a brief description of this research, and a filter question, “Do you have any travel experience in Korea?” was raised. No more questions were posed to those who answered “no”. Next, all of the measurement items for the variables in this study were obtained through a literature review. There were seven variables in this study, which were attitude (AT), face consciousness (FC), conformity (CO), perceived quality of goods (PQG), perceived behavioral control (PBC), shopping intention (SI), and shopping behavior (SB). Each variable had three measurement questions, and most of the measurement questions were used based on the previous scales in use for research and development. Depending on the circumstances, some dimensions needed to be adjusted. Meanwhile, the questionnaire was evaluated using a five-point Likert scale, where “1” meant strongly disagree, “2” meant disagree, “3” meant neutral, “4” meant agree, and “5” meant strongly agree. The 24 items in the questionnaire on the modified theory of planned behavior were based on the existing literature and were appropriately adapted to the topic of this study, as shown in Table 1. The final section investigated respondents’ backgrounds, including their gender, age, education, and monthly income. The establishment of this basic information can provide help in interpreting the results of this study.

### 3.2. Data Collection

The questionnaire for this study was posted on Wenjuanxing (www.wjx.cn, accessed on 4 March 2023), which is a very popular website for designing online questionnaires in China. Since it is simpler to obtain a larger sample size and more replies, the use of online surveys in academic research has gained increasing acceptance [59]. Then, we pasted the final official questionnaire link into two instant messaging and social media applications, QQ and WeChat, which are widely used in China, and we asked users to help us collect responses. At the start of the survey on the Wenjuanxing website, we included a separate question to confirm whether respondents had traveled to Korea and shopped there within the last year. If they selected a negative option, the questionnaire was terminated. The surveys were given out starting from March 2023 to September 2024, and a total of 364 were given out. However, 95 respondents who filled out this survey in under 20 s or who checked a unified response were also disqualified, leading to the final collection of 269 valid surveys with an efficiency rate of 73.9%. Scholars recommend that when using PLS-SEM, the sample size should be at least ten times the number of measurement items across all constructs [60,61]. Accordingly, our sample size was sufficient for conducting PLS-SEM. Table 2 displays the demographic characteristics.

### 3.3. Data Analysis and Techniques

**Figure 1:**
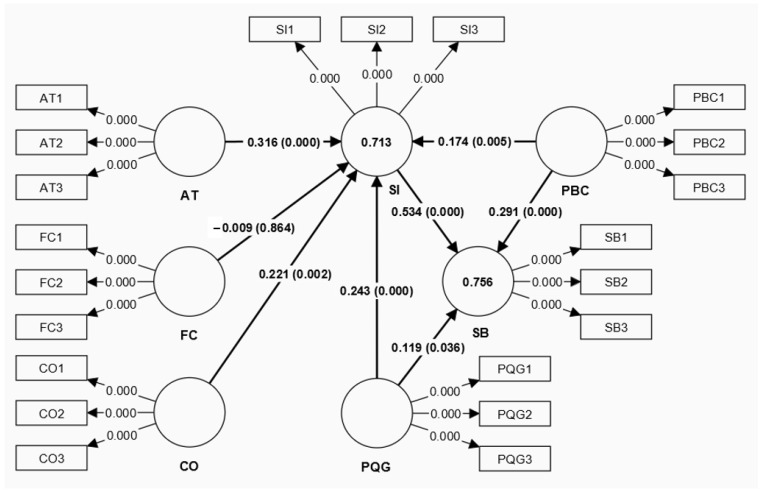
The estimated structural model.

Partial least-squares structural equation modeling (PLS-SEM) is appropriate for exploratory relationship analysis [60,62]. It is an effective method for examining diverse data structures [62]. Studies in the tourism field have applied PLS-SEM in various contexts, including museum marketing [63,64]. Therefore, SPSS v28 was adopted to screen and manage raw data and present the descriptive statistics first. Next, PLS-SEM analysis was conducted in two phases through SmartPLS 4.0: measurement model assessment and structural model assessment [65]. The path coefficient significance was tested using the PLS algorithm and bootstrapping with 5000 subsamples. Figure 1 shows the results of the structural model analysis.

## 4. Results

### 4.1. Measurement Model Assessment

First, an analysis of the partial least-squares structural equation modeling algorithm was used to test the validity and reliability of the measurement model. As Table 3 shows, each item’s loading values were higher than 0.7 (loadings ranging from 0.834 to 0.924), which proved that all items were significantly loaded, and their indicator reliability was verified [60,65].

Internal consistency/reliability can be measured in two ways: composite reliability (CR) and Cronbach’s alpha (α). When the value of Cronbach’s alpha and composite reliability for each item is over 0.7, the internal consistency/reliability of each latent variable can be proved [62,65]. Table 3 shows that Cronbach’s alpha values ranged from 0.806 to 0.893, and the composite reliability values ranged from 0.807 to 0.895.

Then, an evaluation of validity was performed through convergent validity and discriminant validity [65]. According to Hair et al., the average variance extracted (AVE) can be considered a measurement of convergent validity, and each value of the AVE should be over 0.5 [62,66]. As can be seen in Table 3, all latent variables’ AVE values were over 0.7, which indicated good convergent validity.

Next, the discriminant validity was determined by using the Fornell and Larcker criteria [62,67]. As Table 4 shows, all variables’ diagonal values (the square root of the AVE) exceeded the values of any other latent variables underneath them (AT = 0.908, FC = 0.897, CO = 0.849, PQG = 0.895, PBC = 0.883, SI = 0.887, SB = 0.896). Hence, the results conformed to the Fornell and Larcker criteria, and indicated adequate discriminant validity. As a result, the reliability and validity of the measurement model in this study were satisfactory.

### 4.2. Structural Model Assessment

The structural model in this study was assessed through the path coefficients R^2^ (coefficient of determination) and Q^2^ (PLS Predict) [62]. The collinearity assessment in this study was conducted using the variance inflation factor (VIF) before the structural model assessment. As Hair et al. argued, if the value of the VIF is high, the level of collinearity will be higher, which may cause a multicollinearity problem and make the model unreliable [65]. Therefore, when the value of the VIF is ≥5, a collinearity issue may arise. The values of the VIF in this study were all equal to or less than 3.058, which indicated that the model in this study was acceptable and had no collinearity issues.

Afterward, the coefficient of determination (R^2^) was evaluated. R^2^ refers to the explanatory power for all independent constructs [65]. Additionally, as Cohen argued, a value of R^2^ over 0.26 indicates that the explanatory power is substantial [68]. Consequently, the values of R^2^ for shopping intention and shopping behavior were 0.713 and 0.756, respectively, while the values of Q^2^ were 0.697 and 0.729, respectively, indicating the sufficient explanatory power and predictive accuracy of the structural model (Table 5). Meanwhile, Cohen presented a value of f^2^ over 0.35 as an indication of a high effect [68]. Table 6 shows that the highest f^2^ value of 0.426 was observed in the relationship between shopping intention and shopping behavior, which indicated a high impact, and the lowest f^2^ value of 0.023 was observed in the relationship between perceived quality of goods and shopping behavior.

Last, the standardized root mean square residual (SRMR) value of the structural model was 0.054; Henseler et al. argued that if the SRMR value is less than 0.08, the structural model may have a good fit [69]. As a result, the structural model in this study had a favorable model fit. In summary, the structural model in this study had no collinearity problems and had sufficient explanatory power and predictive accuracy.

### 4.3. Hypothesis Testing

To determine the significance of the path coefficients, a bootstrapping approach for PLS-SEM was used [65]. The results obtained after running the bootstrap routine are presented in Table 6. There was a significant relationship between the measured and latent variables when the t-value was >1.96 (*p* < 0.005) at the 95% confidence level (α = 0.05). Conversely, when the t-value was <1.96, there was no relationship between the measured and latent variables. The results showed that attitude (H1: β = 0.316, t = 3.975, *p* = 0.000), conformity (H2-2: β = 0.221, t = 3.093, *p* = 0.002), the perceived quality of goods (H3-1: β = 0.243, t = 3.727, *p* = 0.000), and perceived behavioral control (H4–1: β = 0.174, t = 2.791, *p* = 0.005) had positive relationships with shopping intention. Moreover, the perceived quality of goods (H3-2: β = 0.119, t = 2.098, *p* = 0.036) and perceived behavioral control (H4-2: β = 0.291, t = 4.931, *p* = 0.000) had relationships with shopping behavior. Furthermore, shopping intention (H5: β = 0.534, t = 7.906, *p* = 0.000) also had a significant effect on shopping behavior. Therefore, H1, H2-2, H3-1, H3-2, H4-1, H4-2, and H5 were supported. However, face consciousness (H2-1: β = −0.009, t = 0.172, *p* = 0.864) had no relationships with shopping intention because the t-values of the observed variables were all smaller than 1.96. Meanwhile, the number 0 was contained in the interval of BCI-LL and BCI-UL. Hence, H2-1 was rejected.

## 5. Discussion and Implications

### 5.1. Discussion

The purpose of this study was to investigate which variables affect Chinese tourists’ shopping intentions while shopping in Korea. Specifically, there are four determinants that can affect Chinese tourists’ shopping intentions which are attitude, conformity, perceived quality of goods, and perceived behavioral control. However, this study found that face consciousness failed to have an influence on Chinese tourists’ shopping intentions.

Attitude positively affects Chinese tourists’ shopping intentions. This suggests that a positive attitude toward shopping tourism will positively influence Chinese tourists’ shopping intention for shopping in Korea. This is consistent with previous findings that positive attitudes increase consumer purchase intentions [70]. Additionally, Chinese tourists feel that shopping tourism in Korea is a wise and essential decision, and Chinese tourists are eager to shop during shopping tourism in Korea. The data also imply that Chinese tourists who have a positive attitude toward shopping tourism are more likely to decide whether they are willing to shop. Therefore, the findings reinforce the original theory of planned behavior [70,71].

Conformity positively affects Chinese tourists’ shopping intentions. This suggests that conformity to Chinese-subjective norms will positively influence Chinese tourists’ shopping intentions for shopping in Korea. This is consistent with the claim by Kai et al. that conformity has an important role in shopping intentions, and conformity affects Chinese seniors’ purchase intentions [72]. Meanwhile, this suggests that conformity with Chinese-subjective norms is an important factor influencing Chinese tourists’ willingness to shop, and it provides an important value for local Korean companies in developing shopping tourism marketing programs. Additionally, the data imply that Chinese tourists are willing to shop in Korea while following the relatives or friends surrounding them. Therefore, more attention should be paid to conformity with Chinese-subjective norms when conducting research on the shopping behavior of Chinese tourists in the future. However, Jin and Kang claimed that Chinese consumers’ conformity did not affect purchase intentions toward a US apparel brand, which was inconsistent with our findings. A very important point to note is that their study selected Chinese consumers living in economically developed areas such as Beijing, Shanghai, Guangzhou, etc., whereas the selection of regions for this study included tourists from all regions of China, so the selection was broader and more representative. However, this provides a new direction for our future research on whether these findings are applicable in economically developed cities in China [73]. However, the Chinese-subjective norm of face consciousness did not affect the shopping behavior of Chinese tourists. Outbound tourism is becoming more frequent and convenient in China. Moreover, shopping in Korea has become a common behavior for Chinese tourists. Chinese tourists feel that shopping in Korea does not highlight their worth and position [12]. This suggests that local Korean companies do not need to deliberately focus on the variable of face consciousness when marketing or designing tourism products, but they should rather design tourism products that are more suited to the needs of Chinese tourists.

The perceived quality of goods significantly influences Chinese tourists’ shopping intentions and behavior. This suggests that high-quality goods will positively affect Chinese tourists’ shopping intentions in Korea. At the same time, high-quality items can also directly influence the shopping behavior of Chinese tourists. These data also imply that high-quality goods in Korea are distinctively different from goods made in China, thus frequently attracting the interest of Chinese tourists and increasing their shopping intentions [4]. Hence, Chinese tourists are willing to shop in Korea directly due to the high quality of the goods, and the perceived quality of goods is an extension of the original theory of planned behavior [74].

Perceived behavioral control positively affects Chinese tourists’ shopping intentions and behavior. Qin and Lee argued that it is very convenient for Chinese tourists to shop in Korea, and if they have enough resources (time and funding), they are willing to shop in Korea [75]. This suggests that Korean tourism companies should actively pay attention to providing a good shopping atmosphere to attract Chinese tourists. Additionally, a convenient shopping environment is more attractive to Chinese tourists. Meanwhile, Chinese tourists’ shopping intentions significantly influence their shopping behavior, which is in line with prior studies [76]. The data also imply that positive shopping intentions can greatly boost Chinese tourists’ shopping behavior. These findings highlight the importance of the modified theory of planned behavior for Chinese tourists’ shopping behavior.

### 5.2. Implications

#### 5.2.1. Theoretical Implications

This study offers precious contributions to the existing literature, specifically for the modified theory of planned behavior. The first purpose was to investigate the impacts of attitude, face consciousness, conformity, perceived quality of goods, and perceived behavioral control on shopping intentions.

The results of this study showed relationships between attitude, perceived behavioral control, and intentions, which were consistent with previous theories [77]. Therefore, this result reinforces the existing TPB. Moreover, the findings from this study enhance the understanding of Chinese tourists’ shopping behavior during shopping tourism in Korea [4]. This study also shows that the perceived quality of goods is an important influencing factor for Chinese tourists in improving their shopping behavior during their shopping tourism in Korea [78]. The findings also broaden the current literature on the TPB and strengthen the existing TPB. Although previous studies on shopping behavior mainly focused on Western countries, the findings from this study suggest that the TPB is also suitable for Chinese tourists [71,79].

Lastly, the findings affirmed the reliability and validity of the indicators of attitude, conformity, perceived quality of goods, perceived behavioral control, and intentions. These indicators have implications for the shopping behavior of Chinese tourists. At the same time, these indicators were verified as robust based on existing theories and exhibited statistical significance. Furthermore, these indicators can also provide a solid foundation for future development of the modified TPB [80].

#### 5.2.2. Practical Implications

With the rapid development of shopping tourism in Korea, more and more Chinese tourists choose to go to Korea for shopping tourism. Hence, tourism enterprises need to pay more attention to the aspects of attitude, conformity, perceived quality of goods, and perceived behavioral control of Chinese tourists to attract more of them. The findings from this study have some practical implications for tourism enterprises trying to attract Chinese tourists. Specifically, they are reflected in the following aspects.

First, a positive attitude has a significant impact on Chinese tourists’ shopping intentions. Therefore, tourism enterprises should establish a positive brand image and make efforts to form positive attitude for potential Chinese tourists to gain a good impression through word of mouth [81].

Second, the Korean government improves the perceived behavioral control of Chinese tourists and increases their shopping behavior by reducing shopping tourism costs and increasing the value perceived by Chinese tourists during shopping tourism in Korea [82].

Third, the perceived quality of goods is an important influencing factor for Chinese tourists’ shopping behavior. High-quality Korean goods are more appealing to Chinese tourists, thus increasing their shopping intentions; therefore, enterprises should design high-quality and well-crafted tourism goods according to Chinese preferences [83].

Fourth, Chinese tourists hold the view that face consciousness would not improve their shopping intentions. However, conformity is popular in China; Chinese tourists like to follow the actions of the surrounding people or things [84]. Hence, conformity is an important factor in Chinese tourists’ shopping intentions. Korean tourism companies should develop appropriate marketing strategies to attract Chinese tourists based on conformity.

Lastly, in order to promote the global tourism industry after the COVID-19 epidemic, the Chinese government has launched a permanent visa-free policy with Thailand for ordinary citizens, which facilitates the economic and tourism exchange between the two countries. On the other hand, the Chinese government has implemented short-term visa-free access to Singapore and Malaysia. China has actively created mutual visa-free policies for ordinary citizens of Southeast Asian countries, which shows that China vigorously encourages Chinese people to travel abroad, which will promote the recovery and development of other countries’ economies and tourism. At the same time, China also hopes that the citizens of other countries will travel to China. Many cities in China have implemented visa-free policies for foreigners with 72 or 114 h of transit in order to help China’s economy, the world’s economy, and the tourism market recover. The Chinese government is also continuing to launch a series of favorable policies [85]. Therefore, the Korean government should seize this opportunity to keep attracting Chinese tourists to Korea, and local Korean companies should develop appropriate marketing strategies for Chinese tourists.

## 6. Conclusions, Limitations, and Future Research

This study investigated the factors affecting shopping behavior among Chinese tourists during shopping tourism in Korea. The key findings include the significant impact of attitude, conformity, perceived quality of goods, and perceived behavioral control on shopping intentions, whereas face consciousness did not significantly affect shopping intentions. Furthermore, shopping intentions positively drove shopping behavior.

Despite its contributions, this study has several limitations that need to be noted when interpreting the findings.

First, most of the scale measures utilized in the questionnaire for this study were taken from well-known Western scales, but there was a lack of scales based on the Chinese cultural background in prior literature. Future studies can design more reasonable questionnaire items for the shopping behavior of Chinese tourists. Additionally, experts’ opinions can be sought when designing measurement items to ensure, as much as possible, the comprehensiveness and accuracy of the measurement items.

Second, there are still many factors affecting Chinese tourists’ shopping intentions. In the future, we can think about adding control variables to group Chinese tourists for more in-depth research; for example, we can study whether gender affects shopping intentions and whether there are differences in shopping intentions between young people and older people. In other words, a thorough, in-depth investigation can improve the research model’s accuracy and dependability.

Third, according to the data collected of this study, the target demographic was relatively young people (18–45 years old), making up 81.8% of the sample. The proportion of the elderly was not high, so the results of this study and whether it is representative of the elderly group need to be further verified. Hence, we can consider expanding the method of questionnaire distribution, such as through the use of offline questionnaires, which would be more targeted and allow for a more effective control of the number of young people versus old people. Another option is to work with travel agencies to distribute the questionnaire, which can save time and guarantee the accuracy, completeness, and extensiveness of the data.

## Figures and Tables

**Table 1:** Measurement.

Construct	Item	Source
Attitude (AT)	I think shopping is a necessary activity when traveling in Korea	Han
	I think shopping is a wise choice when traveling in Korea	Taylor and Todd
	I think shopping is good for me when traveling in Korea	[53,54]
Face Consciousness (FC)	While traveling in Korea, shopping can highlight my worth and position	Li et al.
	While traveling in Korea, shopping can make me respected by others	Somogyi et al.
	While traveling in Korea, shopping can allow me to gain respect	[39,55]
Conformity (CO)	I will go shopping if most of my friends go shopping while traveling in Korea	Li et al. Maichum et al.
	I will follow the people with whom I am traveling in Korea to go shopping if they go shopping	
	I will choose the products that my traveling companions like while going shopping in Korea	[39,56]
Perceived Quality of Goods (PQG)	When shopping in Korea, I feel that Korean goods are of good quality	Erdogmus and Budeyri-Turan
	When shopping in Korea, I feel that Korean goods are reliable	
	When shopping in Korea, I feel that Korean goods can be used without worry	Yen [49,57]
Perceived Behavioral Control (PBC)	It is easy to go shopping in Korea if I want	Yadav and Pathak
	Nothing can stop me from shopping in Korea if I want	Han
	I have enough effort (time and money) to go shopping in Korea	[51,53]
Shopping Intention (SI)	I want to go shopping if I have a chance to have a trip to Korea	Li et al.
	I plan to make a trip to Korea for shopping soon	Yen
	I plan to actively save money for shopping in Korea	[39,49]
Shopping Behavior (SB)	I must go shopping during my trip to Korea	Yadav and Pathak
	I go shopping every time I travel to Korea	Perugini and Bagozzi
	Every time I travel to Korea, I buy lots of Korean goods to take home	[51,58]

**Table 2:** Descriptive statistics (*n* = 269).

Construct	Item	Frequency	Proportion
Gender	Male	100	37.2%
	Female	169	62.8%
Age (in years)	<18	11	4.1%
	18–30	131	48.7%
	31–45	89	33.1%
	>45	38	14.1%
Education	High school or below	45	16.7%
	Diploma	32	11.9%
	Bachelor’s degree	108	40.1%
	Master’s degree or above	58	21.6%
	Others	26	9.7%
Monthly income (CNY/Yuan)	3000 or less	74	27.5%
	3001–5000	79	29.4%
	5001–10,000	68	25.3%
	10,001 or more	48	17.8%

**Table 3:** Reliability and validity analysis.

Construct	Item	Factor Loadings	Cronbach’s Alpha	CR	Average Variance Extracted
Attitude (AT)	AT1	0.891	0.893	0.895	0.824
	AT2	0.924			
	AT3	0.908			
Face Consciousness (FC)	FC1	0.901	0.879	0.882	0.804
	FC2	0.888			
	FC3	0.902			
Conformity (CO)	CO1	0.861	0.806	0.807	0.721
	CO2	0.834			
	CO3	0.852			
Perceived Quality of Goods (PQG)	PQG1	0.879	0.876	0.878	0.801
	PQG2	0.901			
	PQG3	0.905			
Perceived Behavioral Control (PBC)	PBC1	0.896	0.859	0.859	0.780
	PBC2	0.889			
	PBC3	0.864			
Shopping Intention (SI)	SI1	0.873	0.865	0.865	0.787
	SI2	0.897			
	SI3	0.891			
Shopping Behavior (SB)	SB1	0.883	0.878	0.878	0.803
	SB2	0.906			
	SB3	0.900			

**Table 4:** Discriminant validity (Fornell).

	AT	FC	CO	PQG	PBC	SI	SB
AT	0.908						
FC	0.646	0.897					
CO	0.753	0.646	0.849				
PQG	0.738	0.676	0.665	0.895			
PBC	0.763	0.558	0.687	0.702	0.883		
SI	0.788	0.599	0.734	0.739	0.732	0.887	
SB	0.830	0.612	0.806	0.719	0.766	0.836	0.896

**Table 5:** Coefficient determination.

Construct	R^2^	Adj. R^2^	Q^2^
Shopping Intention	0.713	0.707	0.697
Shopping Behavior	0.756	0.753	0.729

**Table 6:** Hypothesis testing.

Path	Hypothesis	Path Coefficient	Sample Mean	BCI-LL, BCI-UL	t-Values	f^2^	Decision
AT→SI	H1	0.316	0.309	0.152, 0.462	3.975 ***	0.097	Supported
FC→SI	H2-1	−0.009	−0.008	−0.105, 0.092	0.172	0.000	Rejected
CO→SI	H2-2	0.221	0.224	0.090, 0.365	3.093 **	0.062	Supported
PQG→SI	H3-1	0.243	0.244	0.117, 0.369	3.727 ***	0.072	Supported
PQG→SB	H3-2	0.119	0.118	0.009, 0.232	2.098 *	0.023	Supported
PBC→SI	H4-1	0.174	0.176	0.055, 0.301	2.791 **	0.038	Supported
PBC→SB	H4-2	0.291	0.293	0.179, 0.412	4.931 ***	0.141	Supported
SI→SB	H5	0.534	0.533	0.398, 0.665	7.906 ***	0.426	Supported

*** *p* < 0.001; ** *p* < 0.01; * *p* < 0.05

## Data Availability

The raw data supporting the conclusions of this study will be made available by the authors upon request.

## References

[1] Zhang Z., Schmude J. (2021). Understanding the Travel Constraints of Potential Chinese Tourists Visiting Germany: Experience from Online Travel Community Members. J. China Tour. Res..

[2] UNWTO Tourism Highlights 2019 Edition. https://www.e-unwto.org/doi/pdf/10.18111/9789284421152.

[3] Wijayanti A., Pramezwary A., Putri E.D., Yulianto A., Nurcahyo R., Brahmanto E. (2021). Shopping Tourism Development Through Top Five Products in Yogyakarta City, Indonesia. E-J. Tour..

[4] Panarello D., Gatto A. (2022). Connecting Perceived Service Quality, Value and Shopping Behavior: An Analysis on Chinese College Students Traveling Overseas. Knowledge.

[5] Li X., Lai C., Harrill R., Kline S.F., Wang L. (2011). When east meets west: An exploratory study on Chinese outbound tourists’ travel expectations. Tour. Manag..

[6] Tsang N.K., Lee L.Y., Liu C. (2014). Understanding the Shopping Motivation of Mainland Chinese Tourists in Hong Kong. J. Chi. Tour. Res..

[7] Correia A., Kozak M., Kim S. (2018). Luxury shopping orientations of mainland Chinese tourists in Hong Kong. Tour. Econ..

[8] Kim E., Kim S., Song D. (2013). Study on purchase behavior and satisfaction of Chinese tourists who buy Korean hair cosmetics in Myeong-dong-Targeting women at 20s and 40s. J. Fash. Bus..

[9] Cui Y.H., Choo H.J. (2018). Effect of Home-Host Country Psychic Distance (HHCPD) Perception of Chinese Tourists on Tourism Shopping Motivation and Fashion Brand Attitudes in Korea. J. Korean Soc. Cloth. Text..

[10] KTO Tourism Statistics 2023. https://datalab.visitkorea.or.kr/datalab/portal/nat/getForTourDashForm.do#.

[11] Lee S. (2015). Effects of Korean Wave on Chinese Tourist’s Korean Food Recognition and Purchasing Intention. J. Korean Con. Assoc..

[12] Yoon K.J. (2015). A Study about marketing strategies and tour motivation of Chinese tourists who visit Korea. Asia-Pac. J. Multimed. Serv. Converg. Art Humanit. Sociol..

[13] Seo H., Hwang S.J., Song K. (2014). The Study of Japanese Customers’ Cosmetic Store Satisfaction when Tourists Purchase Korean Cosmetic Products. J. Korean Soc. Costume.

[14] Nguyen P.K., Eun-Soon Y., Ki-Sang R. (2021). Importance-Performance Analysis of Vietnamese Tourists’ Selection Attributes on Shopping Tourism in Korea. J. Tour. Sci..

[15] UNWTO (2024). Global Report on Shopping Tourism, AM Reports.

[16] Nguyen V.T., Choi S. (2022). Effects of shopping tourism choice attributes on tourism satisfaction and behavioral intention of consumers in Korean traditional markets: Focusing on Vietnamese tourists visiting Korea. Int. J. Tour. Hosp. Res..

[17] Xu Y.H., McGehee N.G. (2012). Shopping behavior of Chinese tourists visiting the United States: Letting the shoppers do the talking. Tour. Manag..

[18] Ajzen I. (2011). The theory of planned behaviour: Reactions and reflections. Psychol. Health.

[19] Quintal V.A., Lee J.A., Soutar G.N. (2010). Risk, uncertainty and the theory of planned behavior: A tourism example. Tour. Manag..

[20] Theodorou A., Hatzithomas L., Fotiadis T., Diamantidis A., Gasteratos A. (2023). The Impact of the COVID-19 Pandemic on Online Consumer Behavior: Applying the Theory of Planned Behavior. Sustainability.

[21] Novienthia D. (2022). The Effect of the Korean Wave on Nation Brand South Korea (Study on USU Physics Students). Inspirasi Strateg. J. Kebijak. Publik Bisnis.

[22] Gyoung K.H., Kim Y., Choi A., Sujie W. (2017). A Study for Satisfaction of Chinese Tourists in Korea. J. Digit. Converg..

[23] Hsu C.H., Huang S. (2012). An Extension of the Theory of Planned Behavior Model for Tourists. J. Hosp. Tour. Res..

[24] Park Y., Njite D. (2010). Relationship between Destination Image and Tourists’ Future Behavior: Observations from Jeju Island, Korea. Asia Pac. J. Tour. Res..

[25] Ahn Y., Lee S.K., Ahn Y. (2019). Who Are Domestic Travel Agency Users and Who Buys Full Package Trips? A Study of Korean Outbound Travelers. J. Asian Financ. Econ. Bus..

[26] Liberato D., Liberato P., Silva M. (2020). Shopping Tourism: Comparative Analysis of the Cities of Oporto and Lisbon as Shopping Destinations. Cultural and Tourism Innovation in the Digital Era.

[27] Wong J.M., Law R. (2003). Difference in shopping satisfaction levels: A study of tourists in Hong Kong. Tour. Manag..

[28] Davis L., Qiu X., Davis D. (2017). Chinese tourists’ shopping behavior in the United States. Int. Text. Appar. Assoc. Annu. Conf. Proc..

[29] Timothy D.J. (2014). Trends in Tourism, Shopping, and Retailing. The Wiley Blackwell Companion to Tourism.

[30] Lee S., Jeon S.C., Kim D. (2011). The impact of tour quality and tourist satisfaction on tourist loyalty: The case of Chinese tourists in Korea. Tour. Manag..

[31] Lo A., Qu H. (2015). A theoretical model of the impact of a bundle of determinants on tourists’ visiting and shopping intentions: A case of mainland Chinese tourists. J. Retail. Consum. Serv..

[32] Azhar M., Nafees S., Sujood Hamid S. (2023). Understanding post-pandemic travel intention toward rural destinations by expanding the theory of planned behavior (TPB). Future Bus. J..

[33] Trafimow D. (2009). The Theory of Reasoned Action. Theory Pract..

[34] Seong B., Hong C. (2021). Does Risk Awareness of COVID-19 Affect Visits to National Parks? Analyzing the Tourist Decision-Making Process Using the Theory of Planned Behavior. Int. J. Environ. Res. Public Health.

[35] Han H. (2015). Travelers’ pro-environmental behavior in a green lodging context: Converging value-belief-norm theory and the theory of planned behavior. Tour. Manag..

[36] Fishbein M. (2008). A Reasoned Action Approach to Health Promotion. Med. Decis. Mak..

[37] Bangun C.S., Handra T. (2021). How Theory of Planned Behavior and Percieved Risk Affect Online Shopping Behavior. Aptisi Trans. Manag..

[38] Fishbein M., Yzer M.C. (2023). Using theory to design effective health behavior interventions. Commun. Theory.

[39] Li D.J., Wu B., Wu R.J. (2009). The Model of the Purchase Intention of China’s Consumers. Manag. World.

[40] Zhang C.X., Pearce P., Chen G. (2019). Not losing our collective face: Social identity and Chinese tourists’ reflections on uncivilised behaviour. Tour. Manag..

[41] Qi X. (2011). Face: A Chinese concept in a global sociology. J. Sociol..

[42] Wang W., Wu J., Wu M.Y., Pearce P.L. (2018). Shaping tourists’ green behavior: The hosts’ efforts at rural Chinese B&Bs. J. Destin. Mark. Manag..

[43] Wu J., Wu H.C., Hsieh C.M., Ramkissoon H. (2022). Face consciousness, personal norms, and environmentally responsible behavior of Chinese tourists: Evidence from a lake tourism site. J. Hosp. Tour. Res..

[44] Zhou R., Horrey W.J., Yu R. (2009). The effect of conformity tendency on pedestrians’ road crossing intentions in China: An application of the theory of planned behavior. Accid. Anal. Prev..

[45] Susilawati E., Nova M. (2022). Is Consumptive Behavior Effected by Lifestyle and Conformity? Study at Fashion Community in Sukabumi. J. Econ. Manag. Bus. Account..

[46] Pool G.J., Schwegler A.F. (2007). Differentiating Among Motives for Norm Conformity. Basic Appl. Soc. Psychol..

[47] Lee J.E., Goh M.L., Mohd Noor M.N.B. (2019). Understanding purchase intention of university students towards skin care products. PSU Res. Rev..

[48] Jiang L., Shan J. (2018). Genuine brands or high-quality counterfeits: An investigation of luxury consumption in China. Can. J. Adm. Sci..

[49] Yen Y.-S. (2018). Extending consumer ethnocentrism theory: The moderating effect test. Asia Pac. J. Mark. Logist..

[50] Farooq M.S., Salam M., Rehman S.U., Fayolle A., Jaafar N., Ayupp K. (2018). Impact of support from social network on entrepreneurial intention of fresh business graduates. J. Educ. Train..

[51] Yadav R., Pathak G.S. (2017). Determinants of Consumers’ Green Purchase Behavior in a Developing Nation: Applying and Extending the Theory of Planned Behavior. Ecol. Econ..

[52] Brown M., Pope N.K., Voges K.E. (2003). Buying or browsing? An exploration of shopping orientations and online purchase intention. Eur. J. Mark..

[53] Han T.-I. (2018). Determinants of Organic Cotton Apparel Purchase: A Comparison of Young Consumers in the U.S.A. and South Korea. Sustainability.

[54] Taylor S., Todd P.A. (1995). Understanding information technology usage: A test of competing models. Inf. Syst. Res..

[55] Somogyi S., Li E., Johnson T.E., Bruwer J., Bastian S.E. (2011). The underlying motivations of Chinese wine consumer behaviour. Asia Pac. J. Mark. Logist..

[56] Maichum K., Parichatnon S., Peng K.-C. (2016). Application of the Extended Theory of Planned Behavior Model to Investigate Purchase Intention of Green Products among Thai Consumers. Sustainability.

[57] Erdogmus I.E., Budeyri-Turan I. (2012). The role of personality congruence, perceived quality and prestige on ready-to-wear brand loyalty. J. Fash. Mark. Manag..

[58] Perugini M., Bagozzi R.P. (2001). The role of desires and anticipated emotions in goal-directed behaviours: Broadening and deepening the theory of planned behaviour. Br. J. Soc. Psychol..

[59] Han H., Kim Y. (2010). An investigation of green hotel customers’ decision formation: Developing an extended model of the theory of planned behavior. Inter. J. Hosp. Manag..

[60] Hair J.F., Ringle C.M., Sarstedt M. (2011). PLS-SEM: Indeed a Silver Bullet. J. Mark. Theory Pract..

[61] Lin F., Ryu K. (2024). How does Starbucks’ merchandise design in their online shop trigger behavioral intention?. Int. J. Hosp. Manag..

[62] Hair J.F., Risher J.J., Sarstedt M., Ringle C.M. (2019). When to use and how to report the results of PLS-SEM. Eur. Bus. Rev..

[63] Lin F., Ryu K. (2023). How product design affects repurchase intention, eWOM, and museum visit intention: Museum mystery boxes in China. J. Travel Tour. Mark..

[64] Lin F., Ryu K., Ahn Y.-J. (2024). Effect of product design on repurchase intention, electronic word-of-mouth, and museum visit intention: Museum random boxes in China. Int. J. Tour. Res..

[65] Hair J.F., Sarstedt M., Ringle C.M., Gudergan S.P. (2017). Advanced Issues in Partial Least Squares Structural Equation Modeling.

[66] Ringle C., Silva D., Bido D.D.S. (2014). Structural Equation Modeling with the SmartPLS. Rev. Bras. Mark..

[67] Fornell C., Larcker D.F. (1981). Structural Equation Models with Unobservable Variables and Measurement Error: Algebra and Statistics. J. Mark. Res..

[68] Cohen J. (2013). Statistical Power Analysis for the Behavioral Sciences.

[69] Henseler J., Hubona G.S., Ray P.A. (2016). Using PLS path modeling in new technology research: Updated guidelines. Ind. Manag. Data Syst..

[70] Elmoussaoui A.E., Benbba B. (2023). Determinants of consumer’s online shopping intention during COVID-19. J. Electron. Bus. Digit. Econ..

[71] Wang D., Lee H. (2022). Examining Chinese Tourists’ Revisit Intention in Southeast Asian Countries. Tour. Rev. Int..

[72] Kai Y., Kang Z., Chen Z., Sun X., Tang W. (2021). Social learning? Conformity? Or comparison? An empirical study on the impact of peer effects on Chinese seniors’ intention to purchase travel insurance. Tour. Manag. Perspect..

[73] Jin B., Kang H.J. (2011). Purchase intention of Chinese consumers toward a US apparel brand: A test of a composite behavior intention model. J. Consum. Mark..

[74] Lou X., Chi T., Janke J., Desch G. (2022). How Do Perceived Value and Risk Affect Purchase Intention toward Second-Hand Luxury Goods? An Empirical Study of U.S. Consumers. Sustainability.

[75] Qin F., Lee J.-H. (2014). The Influence of Fashion Product Purchase Criteria and Effects of Store Attributes Toward Shopping Satisfaction for Inbound Chinese Tourist in Korea. Fash. Text. Res. J..

[76] Wen J., Kozak M. (2022). Chinese Outbound Tourist Behaviour.

[77] Prayudhana A., Adrian A., Mardikawanti A., Hendro Y.A., Nugroho N.N. (2022). Examining Determinant Factors on Online Shopping Behavior upon Buying Furniture. Indones. Bus. Rev..

[78] Walia S.B., Kumar H., Negi N. (2020). Impact of brand consciousness, perceived quality of products, price sensitivity and product availability on purchase intention towards ‘green’ products. Int. J. Technol. Manag. Sustain. Dev..

[79] Bui T.H. (2021). Discovering shopping visitors’ behavior and preferences using geo-tagged social photos: A case study of Los Angeles City. J. Mark. Anal..

[80] Hui F., Loahavilai P., Chakpitak N., Chandarasupsang T. (2021). Citespace Knowledge Gap Analysis in Asia Duty Free Tourism Purchasing Behavior. Int. J. Knowl. Eng..

[81] Jin A., Zhang S. (2021). Relationship between South Korea’s National Image, Corporate Image and Brand Image: Based on the Perspective of Chinese Consumers’ Evaluation. Int. J. Front. Sociol..

[82] Ng S.L. (2021). Would you speak softly in public? An investigation of pro-environmental behavior of Chinese outbound tourists in Hong Kong. Curr. Issues Tour..

[83] Song H., Hong J., You Y. (2020). An Analysis of the Differences in Overseas Buyers’ Perception on Korean Consumer Products. Res. World Econ..

[84] Huibin C., Yongqi H., Xingyu P. (2022). Upgraded Sports Consumption Behavior of College Students: Current Characteristics and Influencing Factors. Humanit. Soc. Sci..

[85] Song X. Report Shows: Inbound Tours Shine and Outbound Tours Heat Up. https://baijiahao.baidu.com/s?id=1811307054592260496&wfr=spider&for=pc.

